# Reorganization of three-dimensional chromatin architecture in *Medicago truncatula* under phosphorus deficiency

**DOI:** 10.1093/jxb/erac517

**Published:** 2022-12-27

**Authors:** Tianzuo Wang, Jing Wang, Li Chen, Jiaying Yao, Zan Yuan, Dong Zhang, Wen-Hao Zhang

**Affiliations:** State Key Laboratory of Vegetation and Environmental Change, Institute of Botany, the Chinese Academy of Sciences, Beijing 100093, P. R. China; University of Chinese Academy of Sciences, the Chinese Academy of Sciences, Beijing 100049, P. R. China; State Key Laboratory of Vegetation and Environmental Change, Institute of Botany, the Chinese Academy of Sciences, Beijing 100093, P. R. China; University of Chinese Academy of Sciences, the Chinese Academy of Sciences, Beijing 100049, P. R. China; State Key Laboratory of Vegetation and Environmental Change, Institute of Botany, the Chinese Academy of Sciences, Beijing 100093, P. R. China; University of Chinese Academy of Sciences, the Chinese Academy of Sciences, Beijing 100049, P. R. China; Annoroad Gene Technology (Beijing) Co., Ltd, Beijing 100180, P.R. China; Annoroad Gene Technology (Beijing) Co., Ltd, Beijing 100180, P.R. China; Annoroad Gene Technology (Beijing) Co., Ltd, Beijing 100180, P.R. China; State Key Laboratory of Vegetation and Environmental Change, Institute of Botany, the Chinese Academy of Sciences, Beijing 100093, P. R. China; University of Chinese Academy of Sciences, the Chinese Academy of Sciences, Beijing 100049, P. R. China; Cardiff University, UK

**Keywords:** 3D chromatin architecture, histone modification, hormonal response, *Medicago truncatula*, phosphorus deficiency, root system architecture, transcriptional regulation

## Abstract

Emerging evidence reveals that the three-dimensional (3D) chromatin architecture plays a key regulatory role in various biological processes of plants. However, information on the 3D chromatin architecture of the legume model plant *Medicago truncatula* and its potential roles in the regulation of response to mineral nutrient deficiency are very limited. Using high-resolution chromosome conformation capture sequencing, we identified the 3D genome structure of *M*. *truncatula* in terms of A/B compartments, topologically associated domains (TADs) and chromatin loops. The gene density, expressional level, and active histone modification were higher in A compartments than in B compartments. Moreover, we analysed the 3D chromatin architecture reorganization in response to phosphorus (P) deficiency. The intra-chromosomal *cis*-interaction proportion was increased by P deficiency, and a total of 748 A/B compartment switch regions were detected. In these regions, density changes in H3K4me3 and H3K27ac modifications were associated with expression of P deficiency-responsive genes involved in root system architecture and hormonal responses. Furthermore, these genes enhanced P uptake and mobilization by increasing root surface area and strengthening signal transduction under P deficiency. These findings advance our understanding of the potential roles of 3D chromatin architecture in responses of plants in general, and in particular in *M. truncatula*, to P deficiency.

## Teaser

Reorganization of the 3D chromatin architecture is associated with transcriptional regulation to enhance phosphorus uptake and mobilization in *Medicago truncatula*under phosphorus deficiency.

## Introduction

Chromatin is the main carrier of DNA, which stores the genetic information for living organisms in eukaryotes. Recent developments in spatial genetics have found that the three-dimensional (3D) chromatin architecture plays crucial regulatory roles in diverse biological processes, such as DNA replication and transcription regulation (Grob and Grossniklaus, 2017; Ouyang *et al.*, 2020). Chromatin folding makes different loci containing promoters and enhancers which may be near or far in linear DNA, interact with each other (Dixon *et al.*, 2012; Feng *et al.*, 2014). The 3D chromatin architecture can also expose or hide partial regions to activate or suppress the expression of genes (Zhang and Wang, 2021). The different 3D genome structures are distinguished based on different resolution ratios. Overall, chromosome territories are formed from all chromosomes in the nucleus (Dogan and Liu, 2018), and each chromosome is separated into A and B compartments, which are euchromatic and heterochromatic regions, respectively (P. Dong *et al*., 2017). High-resolution chromosome conformation capture (Hi-C) technology has developed quickly, and can be used to identify smaller structures of the 3D genome (Ouyang *et al.*, 2021). Topologically associated domains (TADs) are relatively independent clusters of chromatin interactions at the sub-megabase scale (Dixon *et al.*, 2012; Sexton and Cavalli, 2015). TADs have been identified in the genomes of most plants, but indistinctly in the Arabidopsis genome (Wang *et al.*, 2015; P. Dong *et al.*, 2017). Generally, one TAD contains chromatin loops with sizes of several kbp (Zhao *et al.*, 2019). Chromatin loops are usually enriched in gene fragments with high levels of expression (Peng *et al.*, 2019; Wang *et al.*, 2021).

The chromatin architecture is not always fixed, and reorganization of the 3D chromatin architecture occurs frequently during the diverse biological processes in plants (Grob and Grossniklaus, 2017; Raxwal *et al.*, 2020). For example, reorganization of the 3D chromatin architecture has been reported to play a role in modulation of the processes associated with domestication (M. J. Wang *et al*., 2017), evolution (Xie *et al.*, 2019), fertilization (Zhou *et al.*, 2019), polyploidization (Wang *et al.*, 2018; Zhang *et al.*, 2019) and tissue development (Y. H. Sun *et al*., 2020). Several studies also reported that chromatin architecture reorganization was involved in the response of plants to environmental changes. Heat-reduced 3D chromatin rearrangement has been found in rice using differential analysis of A/B compartments, TADs and short-range interactions between control and heat treatments (Liang *et al.*, 2021). In Arabidopsis, transposon activation was found to be highly related to 3D chromatin organization under heat stress (L. H. Sun *et al*., 2020). The degree of 3D chromatin reorganization was reported to be relatively small under mildly cool conditions (Liu *et al.*, 2017). However, it is unknown if and how the deficiency of mineral nutrients affects 3D chromatin architecture reorganization.

Phosphorus (P) is one of the essential macronutrients for higher plants. The total P in the soil is abundant. However, the available inorganic phosphate (Pi) to plants is usually low in many types of soils, thus limiting plant growth (T. Z. Wang *et al.*, 2017; Lambers, 2022). About 70% of global arable land is P-deficient (Xu *et al.*, 2019). To cope with P-deficient environments, plants have evolved complex strategies to maximize P uptake and mobilization via morphological, physiological, and molecular changes (Guo *et al.*, 2022). However, no studies have evaluated the effects of P deficiency on 3D chromatin architecture reorganization. Here, we analysed 3D chromatin reconfiguration, histone modification and gene expression in the legume model plant *Medicago truncatula* under P deficiency.

## Materials and methods

### Plant materials and treatments

Seeds of *M. truncatula* ‘Jemalong A17’ were soaked using concentrated sulfuric acid for 7 min, and then rinsed with water. Seeds were sown on wet filter paper to germinate at 26 °C. They were then planted in vermiculite in a greenhouse (26 °C day/22 °C night, and 14 h photoperiod). All seedlings were pre-cultured by watering with full-strength nutrient solution (2.5 mM KNO_3_, 0.5 mM KH_2_PO_4_, 1 mM MgSO_4,_ 0.25 mM CaCl_2_, 100 µM Fe-Na-EDTA, 30 µM H_3_BO_3_, 5 µM MnSO_4_, 1 µM ZnSO_4_, 1 µM CuSO_4_ and 0.7 µM Na_2_MoO_4_) for 4 weeks. Thereafter, half of the seedlings were watered using a reduced KH_2_PO_4_ concentration (1 µM) for 8 d, as phosphate deficient treatment (PD). Samples were collected for physiological index determination, library construction, and sequencing.

### Determination of biomass and concentration of phosphorus

Samples were dried at 80 °C to constant weight, and then dry weight was recorded . Following this, 100 mg dry samples, 5 ml of nitric acid, and 2 ml of hydrogen peroxide were mixed in digestion tubes, and then samples were digested using a microwave acid digestion system (CEM, USA). After diluting and filtering the samples, P concentrations were measured using inductively coupled plasma-atomic emission spectroscopy (Thermo, USA).

### Hi-C sequencing and data processing

Hi-C libraries were constructed using the standard protocol (Belton *et al.*, 2012). About 2 g of leaf samples were crosslinked using 1% formaldehyde to fix the structure of proteins and DNA for each library. Two biological replicates were performed for each treatment. Hi-C libraries were sequenced in an Illumina HiSeq instrument with 2 × 150 bp reads.

After valid-reads were screened using Bowtie2 (--very-sensitive -L 30 --score-min L, –0.6, –0.2 --end-to-end) by mapping reads to the *M. truncatula* genome sequence (Mt4.0; Langmead and Salzberg, 2012); these reads were used to generate raw and ICE (Iterative correction and eigenvector decomposition) normalized matrices at different resolutions using HiC-Pro with default parameters (Servant *et al.*, 2015).

### Identification of A/B compartments, TADs and Loops

ICE-normalized interaction matrices at 20 kb resolution were used to detect A/B compartments as described previously using the Cworld-matrix2compartment module (Lieberman-Aiden *et al.*, 2009). Genome regions with PC1 larger than 0 were defined as A compartments, while the others were B compartments. The expected score within each matrix was calculated using lowes soothed average over the intra-interactions. The observed/expected (OE) ratio was transformed to a final score using the log_2_ function. We calculated the Pearson correlation between patterns of chromosomal interactions of every genome bin, and performed the principle component analysis using a correlation matrix. Positive and negative values of the first principal component were used to separate chromatin regions into A and B compartments, respectively.

ICE-normalized interaction matrices at 10 kb resolution were used to identify TADs by a Perl script Cworld-matrix2insulation method (Lieberman-Aiden *et al.*, 2009). Insulation scores were calculated for each chromosome and were used to identify TAD boundaries (Crane *et al.*, 2015).

We used valid pair reads to call loops using Juicer HICCUPS with default parameters at 5 kb resolution (Durand *et al.*, 2016). After defining loops in each treatment, a merged loop data set was built between two treatments and then the OE value of each loop was calculated for two groups. Differential loops between the two groups were defined with a cutoff as |log_2_Fold change| ≥1. Here, only loops among 2 Mb were used for further analysis. The anchors (5 kb region) which overlapped with gene regions were defined as proximal ones, while those which did not overlap with genes were defined as distal.

### Transcriptome sequencing and analysis

The mRNA was extracted and reverse-transcribed to cDNA for transcriptome sequencing using an Illumina platform with PE150. Three biological replicates were performed for each treatment. Sequenced reads were mapped to the *M. truncatula* genome sequence (Mt4.0) by HISAT2 with default parameters (Kim *et al.*, 2015). We used HTSeq to evaluate the read count of each gene and FPKM (Fragments per Kilobase per Millon Mapped Fragments) to quantify gene expression level (Anders *et al.*, 2015). Differential expression of genes in was analysed by DESeq2 R package with |log_2_Fold change| ≥1 and adjusted *P*<0.05 as a cutoff (Love *et al.*, 2014). The difference in gene density and expression level between A and B compartments was analysed using Wilcoxon unpaired test.

The GO (Gene Ontolog) enrichment of differentially expressed genes was implemented using the hyper geometric test (Fisher’s exact test), in which *P* value was calculated and adjusted. GO terms with adjusted *P*<0.05 were considered to be significantly enriched.

### Chromatin immunoprecipitation sequencing and analysis

About 2.5 g of leaf samples were crosslinked with 1% formaldehyde. Chromatin immunoprecipitation (ChIP) was performed using previously established methods (Wang *et al.*, 2016). Two biological replicates were performed for each treatment. The specific antibodies to trimethylated histone H3 Lys4 (H3K4me3, Abcam-ab8580, USA) and acetylated histone H3 Lys27 (H3K27ac, CST-8173S, USA) were used for ChIP analysis. Library DNA was then purified and amplified for sequencing with an Illumina NovaSeq 6000.

ChIP-seq reads were mapped to *M. truncatula* genome sequence (Mt4.0) using Bowtie2 software (-N 1 -p 6), and only uniquely mapped reads were used to perform downstream analysis (Langmead and Salzberg, 2012). We used DeepTools to generate the correlation plot for all samples. Peak calling was called using MACS2 (Zhang *et al.*, 2008), and then peaks were annotated using BedTools. Reads coverage and depth were calculated by SamTools. Both overlap and MAnorm2 software were used to define differential peaks between two groups (Tu *et al.*, 2021). The visualization of read count data was performed by converting raw bam files to bigwig files using IGV tools. The difference in histone modification levels in A and B compartments was analysed by Wilcoxon unpaired test.

## Results

### Reorganization of the 3D chromatin architecture under P deficiency

To reduce the response to P deficiency, 1-month-old seedlings of *M. truncatula* grown in Pi-sufficient soil (control check, CK) were watered with a Pi-deficient (1 µM P) solution (PD) for 8 d. Exposure of the seedlings to the Pi-deficient medium significantly reduced dry biomass and foliar P concentrations (*P*<0.01), while root/shoot ratio was significantly increased (*P*<0.05) compared with those of CK treatment (Supplementary Fig. S1).

**Fig. 1.**
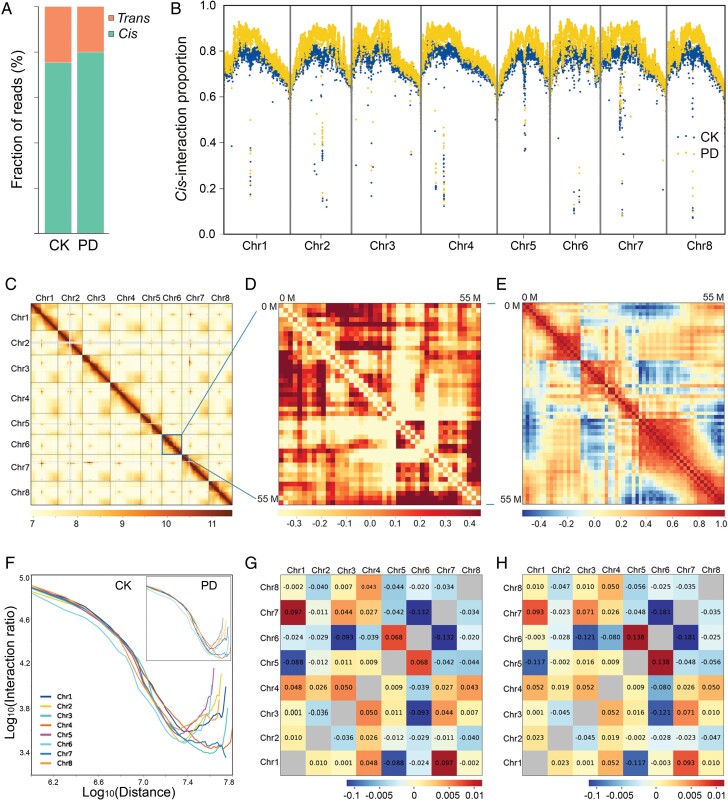
Three-dimensional chromatin architecture of *M. truncatula*. (A) Read fraction of inter-chromosomes (*trans*) and intra-chromosome (*cis*) interactions. (B) *Cis*-interaction proportion along eight chromosomes. (C) Genome-wide Hi-C interaction map of CK at 1 Mb resolution. (D) Iced observed/expected Hi-C interaction map in Chromosome 6 under control check (CK) conditions at 1 Mb resolution. (E) Pearson correlation in Chromosome 6 under CK conditions at 1 Mb resolution. (F) Interaction decay exponents under CK and Pi-deficient (PD) conditions. (G) Inter-chromosomal interactions between all pairs of chromosomes under CK condition. (H) Inter-chromosomal interactions between all pairs of chromosomes under PD conditions.

To explore the differences in the 3D chromatin architecture of *M. truncatula* between the treatment of PD and CK, we obtained a total of 613 Gb and 583 Gb of raw data, respectively, from two biological replicates of Hi-C experiments (Supplementary Table S1). The correlation of chromatin interactions showed that two replicates of Hi-C experiment for the same treatment were highly correlated (Supplementary Fig. S2). Of these data, 622 and 724 million valid paired-end reads were generated for CK and PD, respectively (Supplementary Table S1). Massive valid reads allowed the Hi-C matrix resolution to reach 1 kb (Supplementary Fig. S3), according to the previously established estimation method (Rao *et al.*, 2014). By distinguishing intra- or inter-chromosomal interactions, we found that the proportion of *cis*-interaction was higher than *trans*-interaction (Fig. 1A). Moreover, the proportion of intra-chromosomal *cis*-interaction was enhanced by exposure to P deficiency (Fig. 1A, B). To explore the chromatin interaction, we constructed genome-wide matrix maps (Fig. 1C). Pearson correlations were analysed for each chromosome using the observed/expected Hi-C map, which suggested that the intra-chromosomal interactions of chromosomes 2 and 7 appeared more frequently than the other chromosomes (Fig. 1D, E; Supplementary Fig. S4). For each chromosome, the chromatin interaction frequencies decayed within relatively close range of chromosome distance (Fig. 1F). Chromosomes 1 and 7 had the most inter-chromosomal interaction under the CK treatment, while the most inter-chromosomal interaction was between chromosomes 5 and 6 when exposed to P-deficient medium (Fig. 1G, H).

**Fig. 2.**
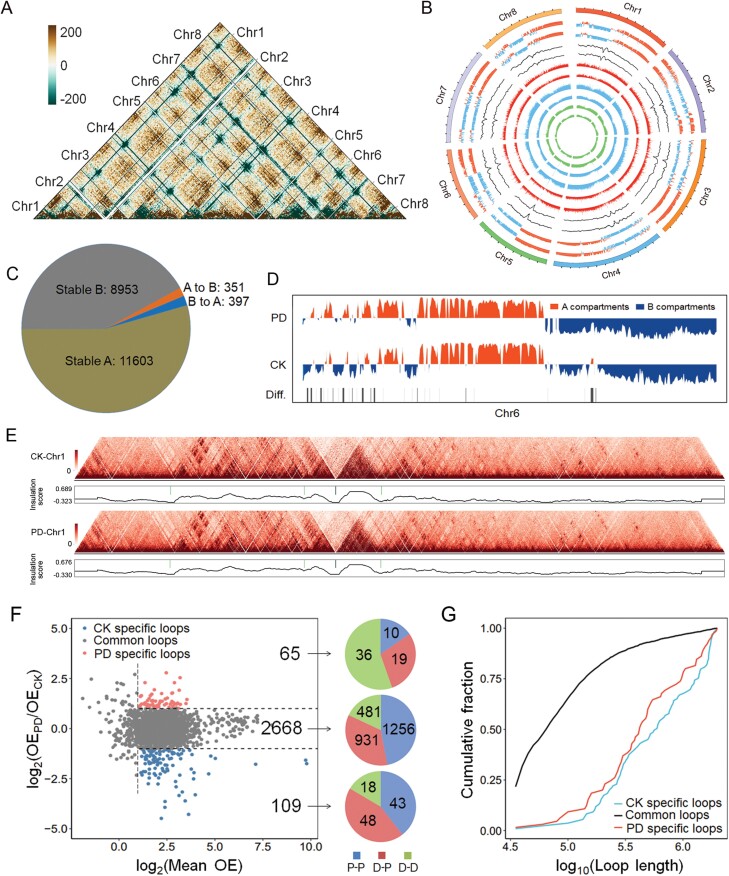
Reorganization of the 3D chromatin architecture under phosphorus deficiency. (A) Hi-C subtraction of Pi-deficient treatment (PD) to control check (CK) conditions in quantile. (B) Circos plot of 3D genome structure, gene expression and histone modification. From outside to inside, circles show A/B compartments of CK, A/B compartments of PD, topologically associated domain (TAD) insulation score of CK, TAD insulation score of PD, gene expression level of CK, gene expression level of PD, H3K4me3 modification of CK, H3K4me3 modification of PD, H3K27ac modification of CK and H3K27ac modification of PD by turn. (C) A/B compartment switch by PD at 20 kb resolution. (D) Difference of Chr6 A/B compartments between CK and PD. (E) TADs and insulation score of Chr1 at 10 kb resolution. (F) Common/specific loops of CK and PD and loop styles. OE: Observed/expected numbers; P-P: Proximal-Proximal; D-P: Distal-Proximal; D-D: Distal-Distal. (G) Length of common/specific loops.

By subtraction analysis of Hi-C heatmap between two treatments, we found that PD induced differences in chromatin interaction (Fig. 2A). Compartments A and B were identified for each chromosome, and the proportion of A compartments was higher than B compartments in the genome of *M. truncatula*. A small scale of A/B compartment switch was found by exposure to Pi-deficient medium (Fig. 2B), which resulted in 351 A to B compartment switch regions, and 397 B to A compartment switch regions in the genome (Fig. 2C). Among the eight chromosomes, chromosome 6 produced the greatest degree of 3D chromatin architecture reorganization under Pi-deficient conditions (Fig. 2D).

We identified the TAD-like regions in the *M. truncatula* genome and found no significant difference between CK and PD (Fig. 2B, E). A total of 2842 loops were identified from the genome of *M. truncatula*. Of them, the observed/expected numbers of 65 and 109 loops were significantly increased and decreased (*P*<0.05), respectively, under PD treatment (Fig. 2F). Among the 65 enriched loops, the proportion of Distal-Distal loops was the highest, but the proportion of this kind of loop was the least among all 2842 loops. In CK-specific loops, 11 genes were up-regulated, and 17 genes were down-regulated under PD treatment. Furthermore, we identified four genes down-regulated in PD-specific loops under PD treatment (Supplementary Table S2). Moreover, the mean length of CK- and PD-specific loops was longer than the common loops (Fig. 2G).

### Transcriptional regulation and chromatin 3D reorganization

**Fig. 3.**
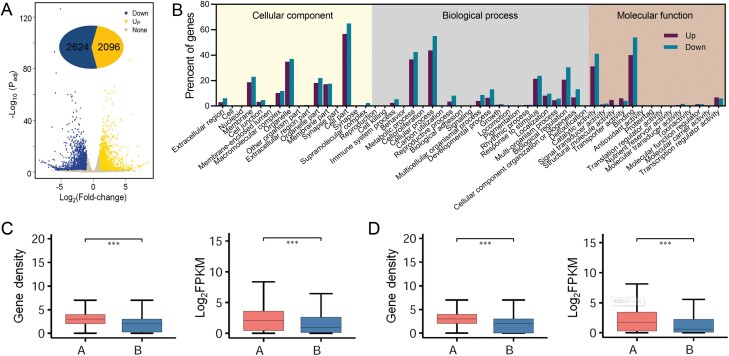
Expression level of differential genes. (A) Number of differential genes showing changes in expression level. (B) GO terms of differential genes. (C) Gene density and expression level between A and B compartments under control check (CK) conditions. (D) Gene density and expression level between A and B compartments under Pi-deficient (PD) conditions. Significant differences at *P*<0.001 using Wilcoxon unpaired test are indicated (***).

Samples treated with the same P deficiency were used to construct cDNA libraries. High-throughput sequencing (RNA-seq) of six libraries generated 41.29 Gb of data (Supplementary Table S3), and the three replicates showed fine correlation (Supplementary Figure S5). P deficiency-responsive genes were identified by comparing the normalized gene expression levels of genes from the PD and CK libraries. We identified 2096 and 2624 genes which were up-regulated and down-regulated, respectively, by the PD treatment (Figs 2B, 3A). The proportion of responsive genes in some secondary GO terms was more than 50%, including organelle, cell part, metabolic process, cellular process, biological regulation, catalytic activity and binding (Fig. 3B).

By conjoint analysis with chromatin architecture, the gene density and expression level in A compartments were significantly higher than those in B compartments both in the CK and PD treatment at *P*<0.001 (Fig. 3C, D). Further analysis identified 33 up-regulated genes in the switch region from B to A compartments, and 40 down-regulated genes in the switch region from A to B compartments in the PD treatment (Supplementary Tables S4, S5).

### Regulation function of histone modification

**Fig. 4.**
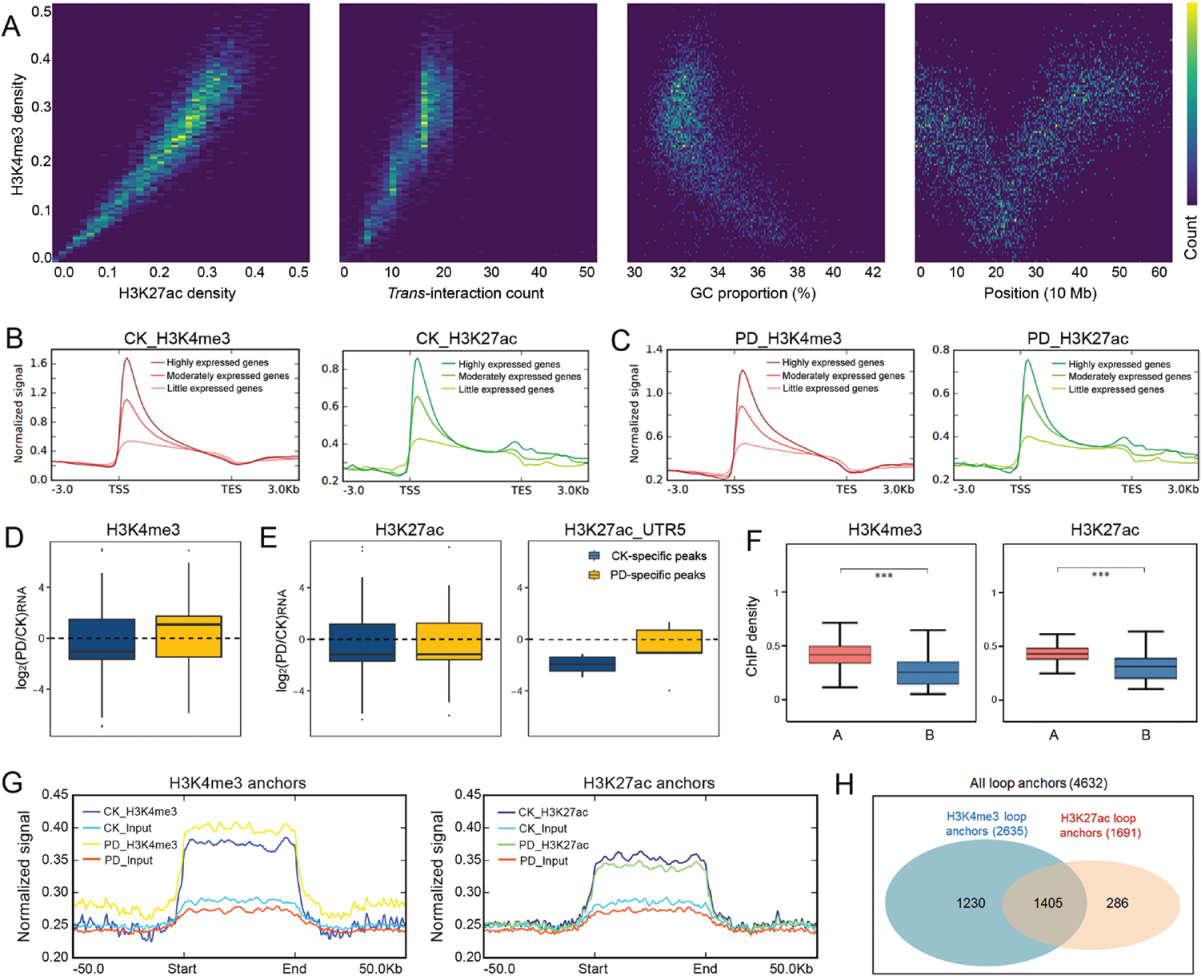
H3K4me3 and H3K27ac modifications. (A) Relationship of histone modification level, *trans*-interaction count, GC proportion and chromosome position. (B) Histone modification and gene expression level in control check (CK) conditions. (C) Histone modification and gene expression level under Pi-deficient (PD) conditions. (D) Phosphorus deficiency-responsive genes and H3K4me3 modification. (E) Phosphorus deficiency-responsive genes and H3K27ac modification. (F) Histone modification level in A and B compartments under CK conditions. Significant differences by Wilcoxon unpaired test at *P*<0.001 are indicated (***). (G) Histone modification in loop anchors. (I) Loop anchors enriched with H3K4me3 and H3K27ac peaks.

Chromatin immunoprecipitation (ChIP) sequencing libraries for H3K4me3 and H3K27ac were constructed using the samples exposed to the same Pi-deficient treatment (Fig. 2B; Supplementary Table S6). Analysis of the two replicates yielded fine correlations (Supplementary Fig. S6). The distribution analysis of histone modification showed that the largest proportion of H3K4me3 and H3K27ac were in UTR5 regions (Supplementary Fig. S7). These two histone modifications exhibited similar enrichment patterns. The histone modification level was positively correlated with *trans*-interaction count, and negatively correlated with the GC proportion of DNA (Fig. 4A). Moreover, the modification density of both H3K4me3 and H3K27ac was positively correlated with the expressional level of genes (Fig. 4B, C). We identified a total of 22 075 and 10 560 peaks for H3K4me3 and H3K27ac, respectively, and specific peaks were identified (Supplementary Fig. S8). Among the PD-responsive genes, the mean fold-change in expression level of genes with PD-specific H3K4me3 peaks was higher than that of genes with CK-specific H3K4me3 peaks, which suggested that PD-specific H3K4me3 peaks contributed to the expression level of PD-responsive genes (Fig. 4D). For H3K27ac peaks, the peaks in the UTR5 regions had similar results as H3K4me3 peaks (Fig. 4E). As the active markers of chromatin, both H3K4me3 and H3K27ac modifications showed more enrichment in A compartments under CK and PD treatment (Fig. 4F; Supplementary Fig. S9). Moreover, H3K4me3 and H3K27ac modifications were enriched in the anchors of loops, with 63% of the loop anchors being modified by either H3K4me3 or H3K27ac, and 30% of those modified by both marks (Fig. 4G, H).

**Fig. 5.**
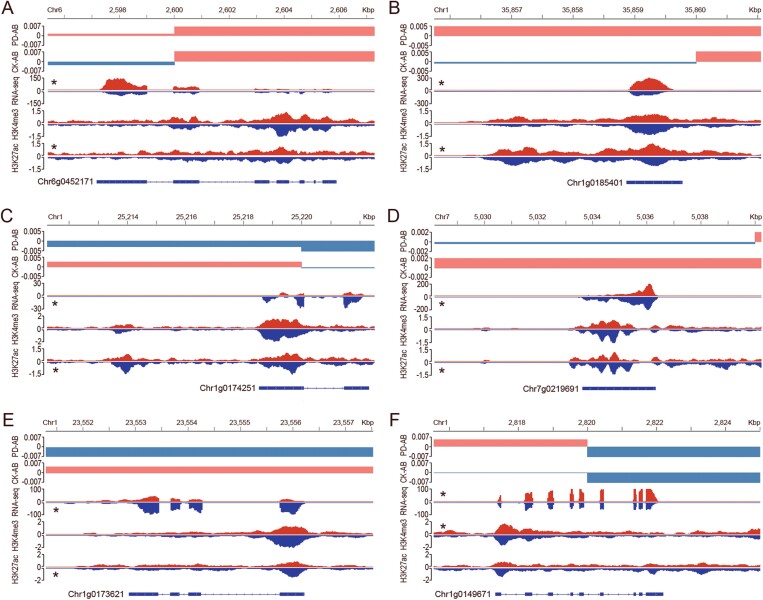
Reorganization of the 3D chromatin architecture, gene expression and histone modification. (A) *Chr6g0452171*. (B) *Chr1g0185401*. (C) *Chr1g0174251*. (D) *Chr7g0219691*. (E) *Chr1g0173621*. (F) *Chr1g0149671*. For A/B compartments, red blocks indicate A compartments, and blue blocks indicate B compartments. For RNA-seq and histone modification, red blocks indicate data under Pi-deficient (PD) conditions, and blue blocks indicate data under control check (CK) conditions. Significantly different gene expression level identified by DEseq or peaks detected by MAnorm2 at *P*<0.05 are indicated (*). At the bottom of each panel, boxes indicate exons, and lines indicate introns of genes.

Here, we have given several examples that showed the correlation of gene expression, A/B compartment switch and histone modifications (Fig. 5). The expression of *Chr6g0452171*, *Chr1g0185401*, and *Chr1g0149671* was up-regulated by P deficiency. Moreover, the location of these genes in chromosomes changed from B compartments to A compartments by treatment with P deficiency. Enriched H3K27ac or H3K4me3 modifications were related to these switches (Fig. 5A, B, F). In contrast, the expression of *Chr1g0174251*, *Chr7g0219691*, and *Chr1g0173621* was down-regulated, which was associated with decrease in histone modifications, and A to B compartment switches after treatment with P deficiency (Fig. 5C, D, E).

## Discussion

Reorganization of the 3D chromatin architecture can generate the switch of euchromatic and heterochromatic regions (A/B compartment switch). It has been reported that 3D chromatin reorganization was associated with histone modification (Tian *et al.*, 2021; Zhao *et al.*, 2019). Before ChIP-seq for histone modification identification, we tested the specificity of as many as eight commercialized histone antibodies to histone of *M. truncatula*, using western blotting. However, only anti-H3K4me3 and H3K27ac antibodies showed good specificity (Supplementary Fig. S10). Therefore, we performed ChIP-seq using these two antibodies. As an active marker, H3K4me3 modification can be identified and bound by effector proteins which activate transcription (Liu *et al.*, 2010). H3K27ac modification of chromatin can loosen chromatin and generally activate transcription (Jiang *et al.*, 2020). This kind of regulatory mechanism has been reported in some biological processes. However, the effect of 3D chromatin architecture reorganization in the response of plants to deficiency in mineral nutrients in general, and in P deficiency in particular, is not known. In the present study, we explored the regulatory mechanisms underlying responses of 3D chromatin architecture reorganization in *M. truncatula* to P deficiency using multi-Omics conjoint analysis of Hi-C, transcriptome, and ChIP-seq. Treatment with P deficiency led to changes in 351 and 397 regions of chromatin from A to B compartments, or from B to A compartments, respectively (Fig. 2C). H3K4me3 and H3K27ac modifications tended to be enriched in A compartments as active markers of transcription (Fig. 4D). Finally, the expression level of some genes was regulated in response to P deficiency.

Remodelling of root system architecture (RSA) is an important way to cope with P deficiency. Plants often increase root surface area by producing more lateral roots and root hairs to increase the Pi uptake under Pi-deficient conditions (Lynch, 2011). Auxin signalling has been found to be closely associated with the modification of RSA under P deficiency (Chiou and Lin, 2011). *Chr6g0452171* encodes an ABC transporter B family protein. This family of proteins is involved in auxin transport, and the homologous gene in Arabidopsis was found to mediate PD-induced RSA remodelling by modulating iron homeostasis (J. S. Dong *et al*., 2017; Kaneda *et al.*, 2011). *Chr1g0174251* is another gene which encodes a leucine-rich repeat protein kinase, and is involved in auxin responses and root hair patterning (Chaudhary *et al.*, 2021; ten Hove *et al.*, 2011). Its homologous gene in Arabidopsis was identified to contain a single nucleotide polymorphism site that was associated with phosphate use efficiency (El-Soda *et al.*, 2019). *Chr7g0219691* encodes a feronia receptor-like kinase which regulates root elongation and responds to stresses (Gigli-Bisceglia *et al.*, 2022; Li *et al.*, 2022). Moreover, this protein family regulates root microbiome regulation to alleviate phosphate starvation (Tang *et al.*, 2022). In our study, the expression level of these three genes was regulated by 3D chromatin architecture reorganization with the alteration of histone modification (Fig. 5A, C, D), which may enhance the tolerance of *M. truncatula* to P deficiency by remodelling RSA and rhizosphere microorganisms.

Abscisic acid (ABA) and Ca^2+^ act as signalling molecules in response to numerous abiotic stresses, including response to P deficiency (Shukla *et al.*, 2021; Zhu *et al.*, 2018). In Arabidopsis, heat shock protein 1 (AtHSP1) and calcium-dependent protein kinase 10 interact to respond to drought stress through ABA and Ca^2+^ signalling (Zou *et al.*, 2010). In our study, the homologous gene of *AtHSP1* in *M. truncatula* was identified in response to P deficiency by regulating 3D genome remodelling (Fig. 5B). The expression of many aldo-keto reductase genes can be regulated by ABA and abiotic stress in *M. truncatula*, and these genes can enhance the tolerance to various abiotic stresses by scavenging cytotoxic aldehydes though the ABA pathway (Yu *et al.*, 2020). Moreover, ribosome biogenesis is an essential process for plants, and some genes involved in this process were found to be responsive to abiotic stress by crosstalk with the ABA signalling pathway in Arabidopsis (Huang *et al.*, 2018; Weis *et al.*, 2015). Here, we found that genes encoding an aldo-keto reductase (*Chr1g0173621*) and a ribosome biogenesis protein (*Chr1g0149671*) were found to be involved in the response to P deficiency (Fig. 5E, F). The mechanisms by which this gene regulates the response to P deficiency warrant further investigation.

In conclusion, we constructed the 3D genome architecture of the legume model plant *M. truncatula*, and revealed that transcriptional regulation was associated with 3D chromatin architecture reorganization under P deficiency. Our findings provide a new perspective to explore the mechanisms underlying the regulation of plant gene transcription in response to P deficiency.

## Supplementary data

The following supplementary data are available at *JXB* online.

Fig. S1. Effects of P deficiency on shoot biomass, P concentrations in shoots and root/shoot ratio.

Fig. S2. Correlation coefficients of four Hi-C libraries.

Fig.S3. Depth in different resolutions.

Fig. S4. Pearson correlation of each chromosome.

Fig. S5. Correlation coefficients of six transcriptome libraries.

Fig. S6. Correlation coefficients of 10 ChIP-seq libraries.

Fig. S7. Distribution of histone modification.

Fig. S8. Common and specific peaks for ChIP-seq of H3K4me3 and H3K27ac.

Fig. S9. Modification level of H3K4me3 and H3K27ac in A and B compartments under PD treatment.

Fig. S10. Western blots using eight commercialized histone antibodies to histone of *M. truncatula*.

Table S1. Statistics of Hi-C sequencing.

Table S2. Different expression of genes located in CK and PD specific loops.

Table S3. Statistics of transcriptome sequencing.

Table S4. Up-regulated genes in the B to A compartment switch regions.

Table S5. Down-regulated genes in the A to B compartment switch regions.

Table S6. Statistics of ChIP sequencing.

erac517_suppl_Supplementary_Figures_S1-S10_Tables_S1-S6

## Data Availability

The sequence data from Hi-C, ChIP-seq and RNA-seq are available at NCBI BioProject under accession number PRJNA874330.
