# Supplementary material for: Reorganization of three-dimensional chromatin architecture in Medicago truncatula under phosphorus deficiency
Source: J Exp Bot. 2022 Dec 27;74(6):2005–15. doi: 10.1093/jxb/erac517 (PMC10049915; doi:10.1093/jxb/erac517)
Supplement: erac517_suppl_Supplementary_Figures_S1-S10_Tables_S1-S6 [file erac517_suppl_supplementary_figures_s1-s10_tables_s1-s6.pdf]

Supporting information

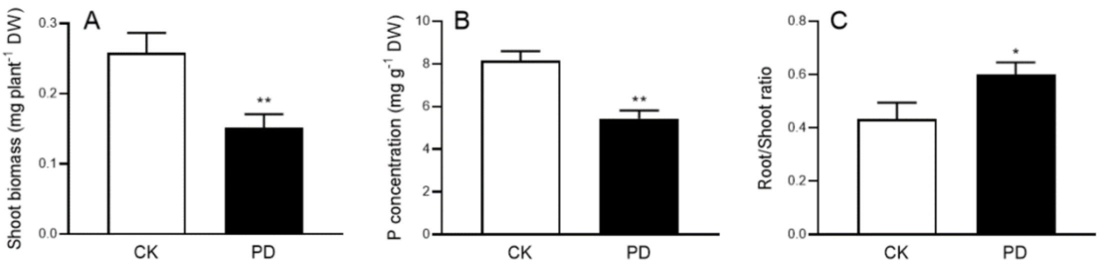

Figure S1. Effects of P deficiency on shoot biomass (A), P concentrations in shoots (B) and root/shoot ratio (C). Data are the means±SE (n=3). Data with "**" and "*" indicate significant different between PD and CK at P<0.01 and P<0.05, respectively. Independent-samples t test is used to analyze differences.

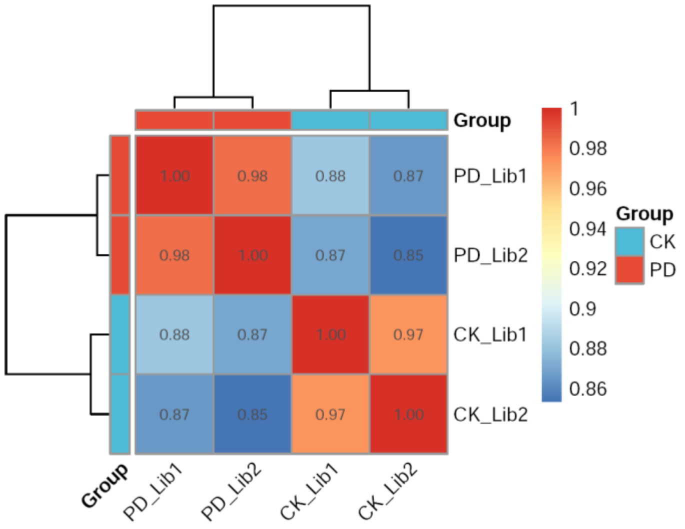

Figure S2. Correlation coefficients of four Hi-C libraries. The stratum-adjusted correlation coefficient heatmap of Hi-C libraries was calculated by Hicrep.

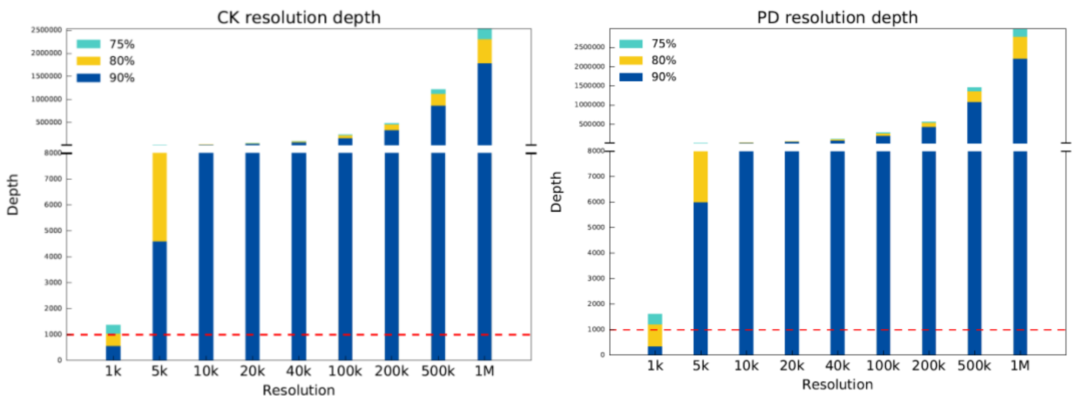

Figure S3. Depth in different resolutions. The matrix resolution of a Hi-C map was define as the smallest locus size such that 80% of loci have at least 1,000 contacts.

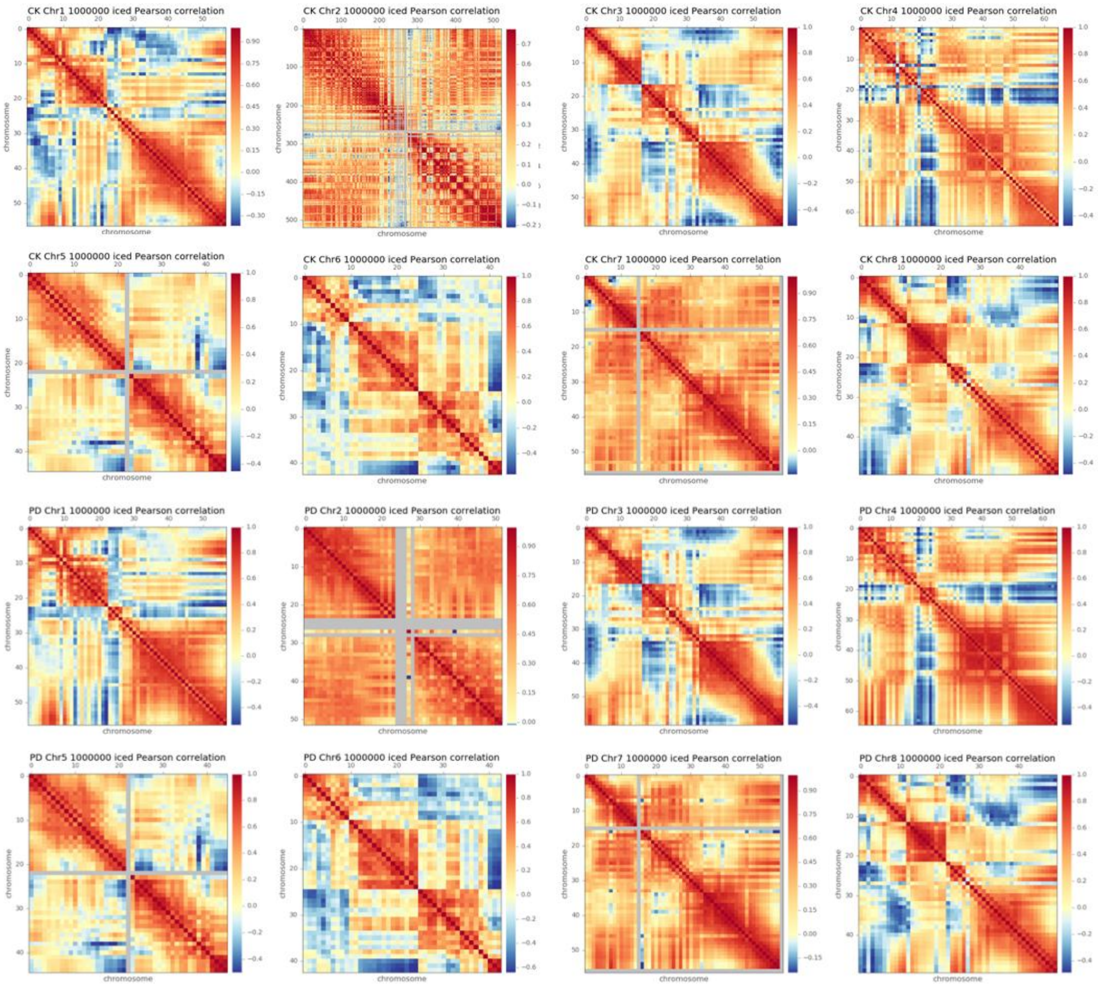

Figure S4. Pearson correlation of each chromosome.

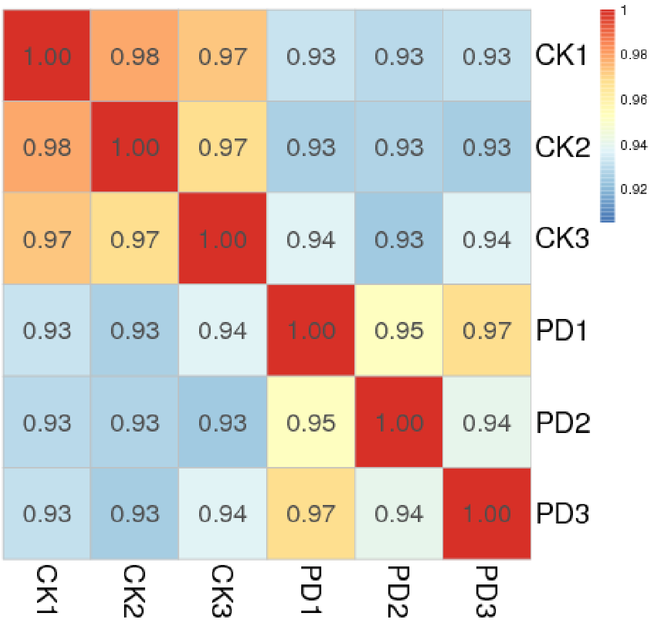

Figure S5. Correlation coefficients of six transcriptome libraries.

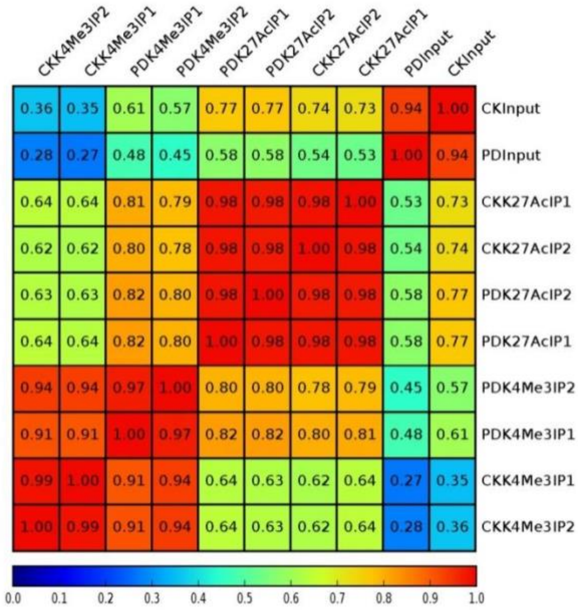

Figure S6. Correlation coefficients of ten ChIP-seq libraries.

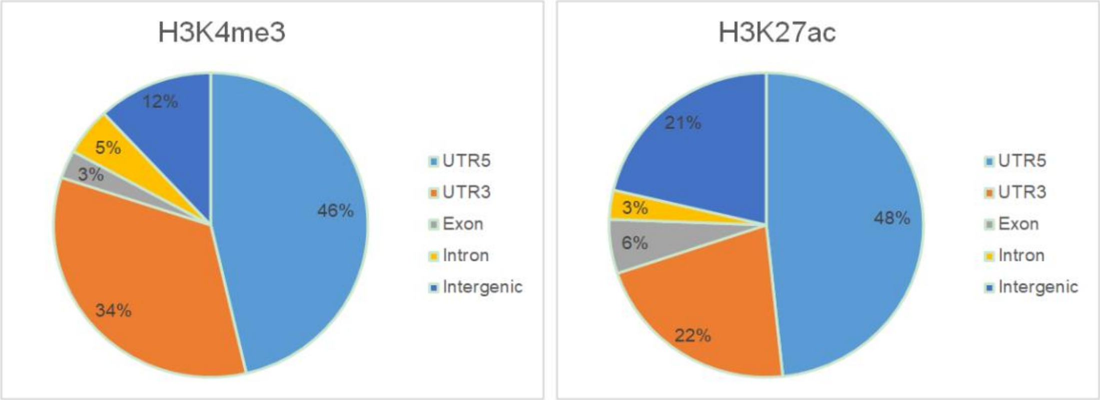

Figure S7. Distribution of histone modification.

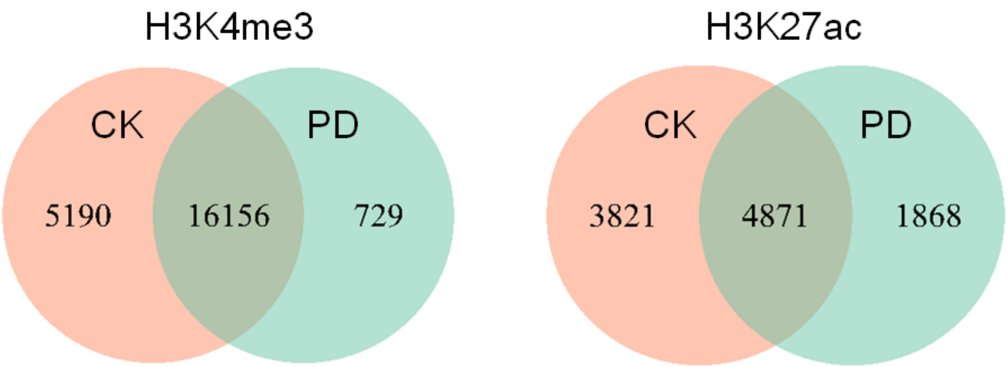

Figure S8. Common and specific peaks for ChIP-seq of H3K4me3 and H3K27ac

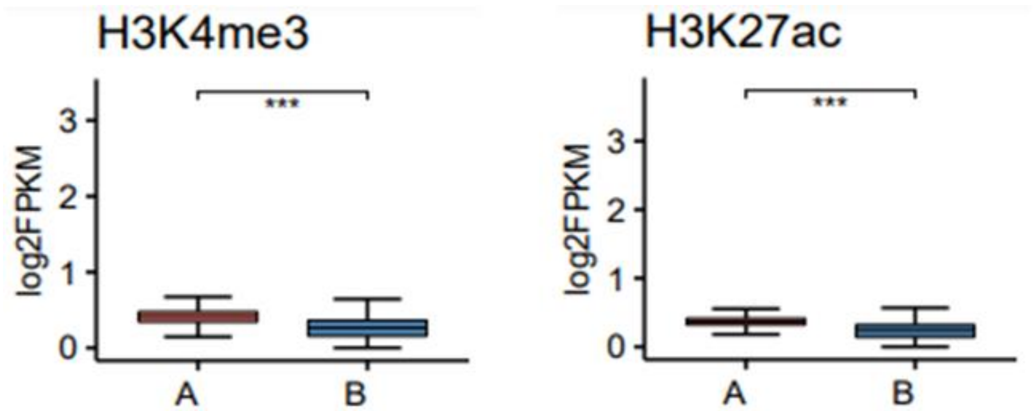

Figure S9. Modification level of H3K4me3 and H3K27ac in A and B compartments under PD treatment. "***" indicates a significant difference at $P<0.001$ using wilcoxon unpair test.

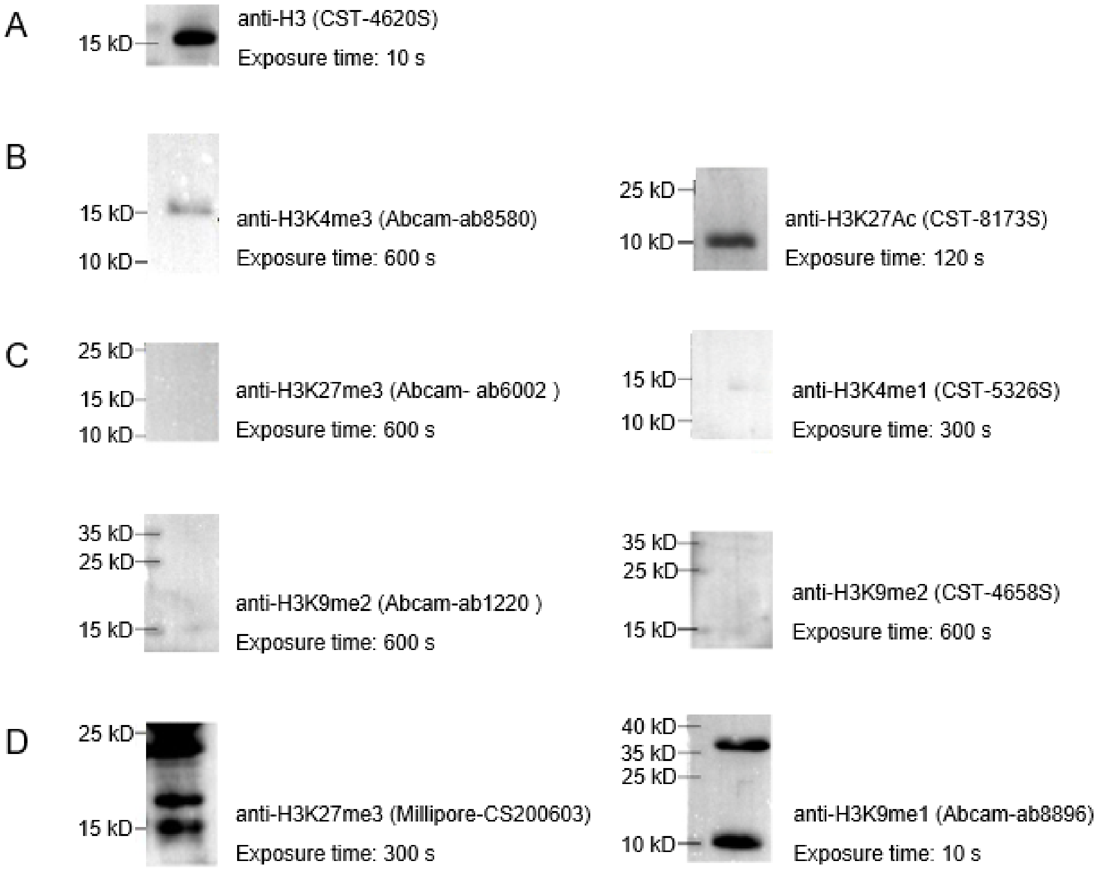

Figure S10. Western blots using eight commercialized histone antibodies to histone of *M. truncatula*. (A) Positive control. (B) Western blots showed good specificity of antibodies to histone of *M. truncatula*. (C) Western blots showed no signal. (D) Western blots showed bad specificity of antibodies to histone of *M. truncatula*. Product number of antibodies and exposure time was shown in pictures.

Table S1. Statistics of Hi-C sequencing.

Sample	CK	PD
Raw Paired-end Reads	2,043,149,515	1,942,863,211
Raw Bases (bp)	612,944,854,500	582,858,963,300
Clean Paired-end Reads	1,945,126,514	1,889,394,093
Clean Paired-end Reads Rate (%)	95.2	97.25
Clean Q30 Bases Rate (%)	92.7	93.31
Unique Mapped Paired-end Reads	880,580,032	1,060,338,741
Unique Mapped Ratio (%)	45.27	56.12
Valid Paired-end Reads	622,346,602	723,943,814
Valid Rate (%)	70.67	68.27

Table S2. Different expressional genes located in CK and PD specific loops.

Loops	Loop types	Different genes	log_2FC	Different expression
Chr5:25730000-26035000	CK specific loop	Chr5g0423841	-3.069573258	down
Chr4:34340000-34535000	CK specific loop	Chr4g0034761	2.512800829	up
Chr7:16555000-18185000	CK specific loop	Chr7g0229421	1.232208594	up
Chr7:19585000-19915000	CK specific loop	Chr7g0230571	3.357507949	up
Chr2:27435000-27660000	CK specific loop	Chr2g0306221	1.450594472	up
Chr8:20285000-20685000	CK specific loop	Chr8g0358201	1.183283763	up
Chr8:37400000-38085000	CK specific loop	Chr8g0375671	-1.773664772	down
Chr4:7490000-7590000	CK specific loop	Chr4g0009651	-2.901593003	down
Chr3:17920000-19675000	CK specific loop	Chr3g0094801	-1.114417188	down
Chr2:755000-1550000	CK specific loop	Chr2g0277801	-1.174518336	down
Chr6:34580000-34915000	CK specific loop	Chr6g0479891	-1.003195462	down
Chr5:34560000-35740000	CK specific loop	Chr5g0434431	2.468248896	up
Chr5:21655000-22480000	CK specific loop	Chr5g0421031	1.021931047	up
Chr3:21605000-21705000	CK specific loop	Chr3g0097031	-4.531028132	down
Chr7:39505000-40025000	CK specific loop	Chr7g0253401	-2.670864225	down
Chr6:16840000-18095000	CK specific loop	Chr6g0468841	1.207706652	up
Chr4:365000-565000	CK specific loop	Chr4g0000901	-1.067621295	down
Chr6:24180000-24315000	CK specific loop	Chr6g0472401	-4.241335456	down
Chr7:4795000-5250000	CK specific loop	Chr7g0219931	-1.324484712	down
Chr5:25730000-26035000	CK specific loop	Chr5g0424111	1.292877834	up
Chr4:34340000-34535000	CK specific loop	Chr4g0035031	-1.937769105	down
Chr6:33730000-33830000	CK specific loop	Chr6g0479191	-1.506767292	down
Chr4:23865000-24055000	CK specific loop	Chr4g0023411	1.396983598	up
Chr8:37400000-38085000	CK specific loop	Chr8g0376551	-1.070786127	down
Chr4:15280000-16655000	CK specific loop	Chr4g0020571	-1.584002098	down
Chr2:15865000-16460000	CK specific loop	Chr2g0298531	1.549198732	up
Chr2:755000-1550000	CK specific loop	Chr2g0278991	-1.162840854	down
Chr3:21605000-21705000	CK specific loop	Chr3g0097121	-1.167991742	down
Chr6:7880000-9435000	PD specific loop	Chr6g0458321	-1.229094994	down
Chr3:30385000-31360000	PD specific loop	Chr3g0107931	-2.720195603	down
Chr4:38980000-39470000	PD specific loop	Chr4g0042001	-2.463082918	down
Chr6:27175000-27325000	PD specific loop	Chr6g0473881	-1.847681088	down

Table S3. Statistics of transcriptome sequencing.

Sample	CK1	CK2	CK3	DS1	DS2	DS3
Raw Reads Number	47,238,972	44,350,066	48,865,872	45,289,390	45,586,134	47,261,388
Raw Bases Number	7,085,845,800	6,652,509,900	7,329,880,800	6,793,408,500	6,837,920,100	7,089,208,200
Clean Reads Number	45,262,056	42,742,494	45,522,222	42,748,726	42,402,540	44,317,524
Clean Reads Rate (%)	95.81	96.38	93.16	94.39	93.02	93.77
Clean Bases Number	6,789,308,400	6,411,374,100	6,828,333,300	6,412,308,900	6,360,381,000	6,647,628,600
Clean Q30 Bases Rate (%)	95.18	95.2	95.19	95.2	95.08	94.72

Table S4. Up-regulated genes in the B to A compartment switch regions.

AB switch	GeneName	Pos	CK.fpkm	PD.fpkm	log_2FC	DE
BA	Chr5g0436701	Chr5:36427184-36429934,-	53.90162	92.9113	1.027201	up
BA	Chr3g0078591	Chr3:1173556-1177014,-	27.20156	46.45792	1.037828	up
BA	Chr8g0365231	Chr8:29405755-29408279,-	2.195493	13.42006	2.862265	up
BA	Chr1g0147101	Chr1:819125-822449,+	18.9163	43.80012	1.436852	up
BA	Chr2g0310301	Chr2:33136327-33141345,-	13.66257	31.5482	1.464937	up
BA	Chr7g0237831	Chr7:27909916-27910144,+	0.081058	3.247624	5.540229	up
BA	Chr7g0222691	Chr7:7951759-7953905,-	0.853482	2.124924	1.527333	up
BA	Chr3g0118951	Chr3:39422943-39424420,-	1.688574	5.945462	2.046502	up
BA	Chr1g0186731	Chr1:36957707-36960032,+	0.007949	0.435999	5.940525	up
BA	Chr8g0346901	Chr8:9917968-9922094,-	9.925207	22.81103	1.448528	up
BA	Chr3g0095661	Chr3:19127721-19131278,-	76.85115	147.9685	1.215783	up
BA	Chr8g0340551	Chr8:4313530-4314402,+	4.625152	11.44796	1.612935	up
BA	Chr1g0185401	Chr1:35858871-35859779,+	7.293963	18.44347	1.578616	up
BA	Chr4g0024101	Chr4:24694268-24694654,-	4.956238	12.30799	1.570408	up
BA	Chr7g0244331	Chr7:33526752-33528596,+	2.561799	5.776483	1.369494	up
BA	Chr6g0469201	Chr6:18874628-18878287,+	5.034545	47.24559	3.463938	up
BA	Chr7g0225391	Chr7:10477121-10483297,-	9.81449	18.0102	1.145846	up
BA	Chr7g0222901	Chr7:8163613-8164494,-	0.885162	1.826965	1.266286	up
BA	Chr8g0349671	Chr8:11951767-11954097,-	12.41618	36.16461	1.773261	up
BA	Chr8g0349751	Chr8:12028349-12031963,-	0.190861	0.873683	2.425388	up
BA	Chr1g0160791	Chr1:11153270-11157474,-	24.35108	41.22905	1.006278	up
BA	Chr1g0188641	Chr1:38339473-38340237,+	0	0.617506	5.696137	up
BA	Chr2g0319461	Chr2:40884879-40885670,+	44.05283	76.73134	1.063705	up
BA	Chr4g0053511	Chr4:47451364-47452235,+	0.223852	0.710911	1.900784	up
BA	Chr7g0221181	Chr7:6687462-6690577,+	24.46691	47.19003	1.196401	up
BA	Chr4g0037021	Chr4:35677773-35681358,-	15.95995	28.58364	1.085943	up
BA	Chr2g0325151	Chr2:45138033-45140965,-	99.02396	217.0868	1.382235	up
BA	Chr1g0150001	Chr1:3095481-3102703,+	1.09914	2.002585	1.120613	up
BA	Chr1g0159911	Chr1:10512905-10513668,+	1.275917	4.073029	1.930875	up
BA	Chr4g0054171	Chr4:47978105-47980540,-	7.466871	18.06382	1.513931	up
BA	Chr8g0349741	Chr8:12007171-12009649,-	4.364418	7.323169	1.013226	up
BA	Chr5g0435721	Chr5:35732695-35735726,+	94.16037	162.1583	1.014578	up
BA	Chr4g0044171	Chr4:41036056-41038313,-	0.228265	0.589082	1.658481	up

Table S5. Down-regulated genes in the A to B compartment switch regions.

AB switch	Gene Name	Pos	CK.fpkm	PD.fpkm	log_2FC	DE
AB	Chr8g0367101	Chr8:30854574-30862557,+	0.410767	0.079066	-2.06795	down
AB	Chr3g0117611	Chr3:38476228-38543691,+	0.854648	0.189305	-1.94059	down
AB	Chr3g0117611	Chr3:38476228-38543691,+	0.854648	0.189305	-1.94059	down
AB	Chr7g0214731	Chr7:858565-862744,-	17.37132	4.068338	-1.82455	down
AB	Chr8g0334621	Chr8:294279-314848,-	13.08443	1.192545	-3.25676	down
AB	Chr4g0004091	Chr4:3110877-3114467,+	29.7826	12.05095	-1.11288	down
AB	Chr2g0299881	Chr2:17733581-17741372,+	1.884278	0.665782	-1.29353	down
AB	Chr1g0173371	Chr1:22982277-23007676,-	3.787778	1.523966	-1.05651	down
AB	Chr8g0359881	Chr8:23657812-23662361,+	0.210787	0.01957	-3.20059	down
AB	Chr6g0480421	Chr6:35196153-35201556,+	0.454436	0.131265	-1.53122	down
AB	Chr5g0444961	Chr5:42213996-42218113,-	5.061449	2.053008	-1.09318	down
AB	Chr8g0359891	Chr8:23662852-23702783,+	0.455651	0.102683	-1.84282	down
AB	Chr1g0174111	Chr1:24815923-24826415,-	1.232101	0.468221	-1.16008	down
AB	Chr1g0153511	Chr1:5976337-5982307,-	0.507265	0.048217	-3.14881	down
AB	Chr1g0176611	Chr1:28139894-28144613,-	5.041183	1.389716	-1.63672	down
AB	Chr3g0085091	Chr3:6430353-6436074,-	1.527719	0.401969	-1.7022	down
AB	Chr8g0337031	Chr8:2053428-2068302,-	17.72887	7.388819	-1.04905	down
AB	Chr8g0353431	Chr8:15232000-15236342,-	4.172031	1.664812	-1.11405	down
AB	Chr4g0008911	Chr4:6885731-6889763,+	1.174794	0.2419	-1.9583	down
AB	Chr5g0442381	Chr5:40550589-40554873,+	63.35991	17.64313	-1.61729	down
AB	Chr6g0482411	Chr6:37081604-37086454,-	3.957676	1.172234	-1.5317	down
AB	Chr4g0004281	Chr4:3339969-3353379,-	9.165706	3.712572	-1.09952	down
AB	Chr2g0299881	Chr2:17733581-17741372,+	1.884278	0.665782	-1.29353	down
AB	Chr8g0342071	Chr8:5485180-5490579,-	6.245932	1.143442	-2.14198	down
AB	Chr4g0012991	Chr4:10386925-10388117,+	388.4117	133.7873	-1.22449	down
AB	Chr1g0174251	Chr1:25218538-25222323,+	1.376464	0.420352	-1.46799	down
AB	Chr2g0304071	Chr2:21892472-21898557,+	0.246714	0.071694	-1.57391	down
AB	Chr5g0443441	Chr5:41252476-41262814,-	2.501463	0.962761	-1.16281	down
AB	Chr5g0443411	Chr5:41238268-41240929,+	27.12265	3.257776	-2.82454	down
AB	Chr6g0482391	Chr6:37068853-37071779,-	3.800218	1.127934	-1.50194	down
AB	Chr1g0148891	Chr1:2226324-2227621,+	0.244342	0	-4.52207	down
AB	Chr1g0148921	Chr1:2235716-2246160,+	2.850743	1.155132	-1.07012	down
AB	Chr1g0173621	Chr1:23552892-23556235,-	9.482753	3.62292	-1.1922	down
AB	Chr1g0146931	Chr1:659056-663705,-	4.800815	1.777113	-1.17278	down
AB	Chr2g0308321	Chr2:31177008-31182049,+	2.868358	0.681054	-1.83394	down
AB	Chr5g0444891	Chr5:42177905-42181401,+	21.50368	9.303323	-1.0092	down
AB	Chr3g0093881	Chr3:16124819-16127868,-	0.469166	0.089018	-2.1901	down
AB	Chr8g0377871	Chr8:39064272-39065471,-	1.260678	0.311908	-1.7765	down
AB	Chr1g0174111	Chr1:24815923-24826415,-	1.232101	0.468221	-1.16008	down
AB	Chr7g0219691	Chr7:5033629-5036331,+	6.907554	1.872129	-1.65957	down

Table S6. Statistics of ChIP sequencing.

Sample	Raw Bases Number	Clean Bases Number	Clean Reads Rate (%)	Clean Q30 Bases Rate (%)
CK-Input	6,144,372,300	5,823,888,300	94.78	93.98
CK- H3K4Me3-IP1	7,970,291,400	7,615,233,000	95.55	94.28
CK- H3K4Me3-IP2	8,122,177,800	7,792,500,600	95.94	94.2
CK- H3K27Ac-IP1	6,850,757,100	6,522,323,400	95.21	94.58
CK- H3K27Ac-IP2	5,863,258,800	5,622,275,100	95.89	94.25
PD-Input	7,394,309,700	6,935,657,700	93.8	93.33
PD-H3K4Me3-IP1	5,687,494,500	5,535,060,300	97.32	93.67
PD- H3K4Me3-IP2	6,610,273,500	6,331,425,300	95.78	93.81
PD- H3K27Ac-IP1	6,848,766,600	6,562,378,500	95.82	94.13
PD- H3K27Ac-IP2	6,454,937,400	6,233,886,300	96.58	93.87
